# Prevalence of helminthic infections and determinant factors among pregnant women in Mecha district, Northwest Ethiopia: a cross sectional study

**DOI:** 10.1186/s12879-018-3291-6

**Published:** 2018-08-06

**Authors:** Berhanu Elfu Feleke, Tadesse Hailu Jember

**Affiliations:** 1 Department of Epidemiology and Biostatistics, University of Bahir Dar, Bahir Dar, Ethiopia; 2 College of Medicine and Health Sciences, Bahir Dar University, Bahir Dar, Ethiopia

**Keywords:** Prevalence, Determinants, Intestinal parasite, Pregnant women, Ethiopia

## Abstract

**Background:**

Intestinal parasites are the most common infections in developing countries. Prevalence and impacts of these parasites are high in pregnant women. The aims of this study were to determine prevalence of helminthic infection and evaluate the determinant factors during pregnancy.

**Methods:**

A cross-sectional study was conducted in Mecha district from November 2015 to January 2016. The data were collected by interview technique and collecting the stool sample from each pregnant woman. Descriptive statistics and binary logistic regression were used.

**Results:**

A total of 783 pregnant women were included. The prevalence of intestinal parasite among pregnant women was 70.6% [95% CI 67 -74%]. *Ascaris lumbricoides* (32.7%) was the predominant intestinal parasite species. Intestinal parasitic infection were 2.94 folds higher in the absence of latrine (AOR: 2.94 [95% CI: 1.5–5.8]). Absence of regular hand washing habit increase the odds of infection by 3.33 folds higher (AOR: 3.33 [95% CI: 1.54–7.14]). Not wearing shoe increased the odds of helminthic infection by 6.87 folds higher (AOR: 6.87 [95% CI: 3.67–12.9]). Illiteracy increases the odds of intestinal parasitic infection by 2.32 folds higher (AOR: 2.32 [95% CI: 1.04–5.26]). Ingestion of raw vegetables increases the odds of intestinal parasitic infection by 2.65 folds higher (AOR: 2.65 [95% CI: 3.23–9.9]). The odds of intestinal parasitic infection were higher in rural areas (AOR: 2 [95% CI: 5–10]). Intestinal parasitic infection was higher in women aged less than 21 years (AOR: 6.48 [95% CI: 2.91–14.4]).

**Conclusion:**

The prevalence of helminthic infection is high in this study. Latrine utilization, hand washing habit, eating raw vegetables and bare foot were the major determinant factors for the high prevalence. Therefore, health education and improvements in sanitary infrastructure could achieve long-term and sustainable reductions in helminth prevalence.

## Background

Intestinal parasites especially geohelmenths are the most common and widespread of human parasites in the developing world [1]. Thousands of rural and impoverished villagers are often chronically infected with different species of parasitic worms [2]. More than 1.5 billion people, or 24% of the world’s population, are infected with soil-transmitted helminthic infections worldwide. Infections are widely distributed in tropical and subtropical areas, with the greatest numbers occurring in sub-Saharan Africa, the Americas, China and East Asia [3].

Intestinal parasitic infection is very common in Ethiopia [4] and the magnitude of infection varies from place to place [5]. Intestinal parasitic infections account the second most predominant causes of outpatient morbidity in the country [6]. High prevalence of parasitic infection in Ethiopia were due to the unsafe and inadequate provision of water, unhygienic living conditions, the absence of proper utilization of latrine and habit of walking with a bare foot [7, 8].

Pregnant women are also at high risk of parasitic infection due to their close relationship with children [9]. Recently, a study done on pregnant women indicated that pregnancy has been associated with an increasing prevalence of parasitic infections compared to non-pregnant women [10].

Infections with helminth were associated with a modest decrease in hemoglobin levels and indicators of poor nutritional status. Helminthic infections, such as Hookworm, Trichuriasis, and Schistosomiasis, have been shown to directly contribute to severe anemia in patients through blood loss and micronutrient deficiencies [11]. Low hemoglobin level is associated within areas where with a high prevalence of Hookworm infection [12]. Hookworm is the leading cause of pathologic blood loss in endemic areas [13].

Anemia accounts 20% of maternal death globally [14]. Anemia in these highly endemic regions is common among pregnant women and often multi-factorial. Anemia has a devastating effect on pregnant women and has been associated with stillbirth, prematurity and low birth weight [15].

Although there are a lot of factors that causes anemia, intestinal parasites like Hookworm, *Trichuriasis trichuira* and schistosoma are highly associated to cause anemia in pregnant women in endemic parts of Ethiopia. These parasites cause anemia directly by feeding the red blood cells or indirectly by causing bleeding, feeding the micronutrients and infiltrating the blood forming organs. The complication of intestinal parasitic infection during pregnancy leads to stillbirth, prematurity and low birth weight. To minimize the burden of parasitic infection during pregnancy, studying the prevalence of intestinal parasitic infections in pregnant women is very ideal. Therefore, the aims of this study were to determine the prevalence and determinants of intestinal helminth among pregnant women in Northwest Ethiopia.

## Methods and materials

A cross sectional study was conducted in Mecha district. Mecha district was located 40 km to the north of Bahir dar city and the district contains 376,000 residents. The data were collected from November 2015 to January 2016. The target population was all pregnant women residing in Mecha district. Pregnant women absent during the data collection period or unable to give stool sample were excluded from the study.

The sample size was calculated using single population proportion formula with the assumption of 95% CI, 50% proportion of intestinal parasite in pregnant women, 5% margin of error, none response rate of 10% and a design effect of 2 gives 846 pregnant women’s. Multistage sampling technique was used. First 10 kebeles (the smallest administrative unit in Ethiopia) were selected from 40 kebeles of Mecha district using simple random sampling technique. Then simple random sampling technique was used to select pregnant women from these 10 kebeles using the kebele health extension workers registration list as a sampling frame.

The data collection procedure was conducted using interview technique and collecting stool sample from each interviewed pregnant women. Fifteen health extension workers were recruited for the data collection and 5 clinical nurses were recruited for supervision. The stool sample was collected from each pregnant woman, preserved with 10 ml sodium acetate- acetic acid-formalin solution (SAF) and transported to Bahir dar regional laboratory for analysis. From each pregnant woman, one gram stool sample was collected. Concentration technique was used. The stool sample was well mixed and filtered using a funnel with gauze then centrifuged for one minute at 2000 RPM (revolution per minute) and the supernatant was discarded. 7 ML (Milliliter) normal saline was added, mixed with a wooden stick, 3 ML ether was added and mixed well then centrifuged for 5 min at 2000 RPM. Finally, the supernatant was discarded and the whole sediment was examined for parasites [16].

To ensure the quality of this research, training was given for all data collectors and supervisors. The pre-test was conducted in 50 pregnant women then the necessary correction was done on the questionnaire after the pre-test. The whole data collection procedures were closely supervised by field supervisors and investigators.

We say the women practice proper hand hygiene if she washed her hands after visiting the toilet, before cooking food and before feeding her child. We say the women properly utilize toilets if the household members consistently utilize the toilets.

The data were entered into the computer using Epi-info software and transported to SPSS software for analysis. Descriptive statistics were used to estimate the prevalence of intestinal parasite and binary logistic regression was used to identify the determinants of intestinal parasite and variable with a *p*-value less than 0.05 was declared as determinants of intestinal parasite. Adjustments were done for age, gravidity, parity, ANC visit, religion, ethnicity, residence, ingestion of raw vegetables, latrine utilization, hand washing practice, bare foot and educational status.

## Results

A total of 783 pregnant women were included giving a response rate of 92.55%. The mean age of the responders was 20.3 years (Standard deviation [SD] 2.95 years). The majority (91.8%) of respondents were orthodox Christian by religion. Amhara ethnicity constituted 87.9% of the study participants (Table 1).

**Table 1:** Socio-demographic profile of pregnant women in Mecha district, Northwest Ethiopia (*n* = 783)

SN^a^	Population profile		N^b^	Percentage
1.	Age	16–20	489	62.5
		21–25	291	37.2
		> 25	3	0.4
2.	Residence	Urban	181	23.1
		Rural	602	76.9
3.	Religion	Orthodox	719	91.8
		Muslim	64	8.2
4.	Ethnicity	Amhara	688	87.9
		Agaw	74	9.5
		Others	21	2.6
5.	Educational status	Illiterate	194	24.5
		Elementary	458	58.5
		Secondary	36	4.6
		Certificate	43	5.5
		Diploma	16	2
		First degree	35	4.5
		Second degree	1	0.1
6.	Gravidity	1	263	33.6
		2	329	42
		3	185	23.6
		4	6	0.8
7.	Frequency of ANC visit	0	275	35.1
		1	314	40.1
		2	189	24.1
		3	5	0.6
8.	Gestational age in weeks	14–23	157	20.1
		24–33	293	37.4
		34–42	333	42.5

^a^*SN* serial number

^b^*N* number of women

The prevalence of intestinal parasite among pregnant women was 70.6% [95% CI 67 -74%].

60.8% of the infection was on high intensity of infection, 18.3% of the infection was on moderate intensity of infection and 21% of the infection was on low intensity of infection (Table 2).

**Table 2:** The helminthic infection status of pregnant women

Parasites	Number of women infected	Percentage of women infected
Ascaris lumbricoides	256	32.7
*Schistosoma mansoni*	136	17.4
Hookworm	111	14.2
Strongyloides stercolaris	50	6.4
Mixed infection	42	5.36

The absence of latrine increases the odds of intestinal parasitic infection by 2.94 folds higher. The odds of intestinal parasitic infection were 3.33 folds higher among pregnant women that didn’t have regular hand washing habit. Not wearing shoe increases the odds of intestinal parasitic infection by 6.87 times higher. Illiteracy increases the odds of intestinal parasitic infection by 2.32 folds higher. The odds of helminthic infections were 2.65 times higher in pregnant women that ingest raw vegetables. Pregnant women whose age less than or equal to 21 years old were 6.48 times more likely to be infected with helminth than the others. The odds of intestinal parasitic infections were 2 folds higher in the rural areas (Table 3).

**Table 3:** Determinants of helminthic infection among pregnant women in Mecha district, Northwest Ethiopia (n = 783)

Variables		^a^IP Infected	IP Not infected	^b^COR[95%CI]	^c^AOR[95%CI]	*p*-value
Latrine	Absent	136	24	2.8 [1.72–4.58]	2.94 [1.5–5.8]	< 0.01
	Present	417	206			
Hand washing habit	Absent	182	15	7.03 [3.94–12.74]	3.33 [1.54–7.14]	< 0.01
	Present	371	215			
Residence	Rural	530	72	50.57 [29.82–86.4]	2 [5–10]	0.01
	Urban	23	158			
Bare footed	Yes	199	105	0.67 [0.48–0.93]	6.87 [3.67–12.9]	< 0.01
	No	354	125			
Educational status	Illiterate	135	59	0.94 [0.65–1.35]	2.32 [1.04–5.26]	< 0.01
	Literate	418	171			
Habit of eating raw vegetables	Yes	426	142	2.08 [1.47–2.94]	2.65 [3.23–9.9]	< 0.01
	No	127	88			
Age in years	<= 21	179	42	2.44 [1.44–3.19]	6.48 [2.91–14.4]	< 0.01
	> 21	374	188			

^a^IP=intestinal parasites

^b^Crude odds ratio

^c^Adjusted odds ratio

## Discussion

The prevalence of helminthic infection was 70.6% (95% CI: 67 - 74%). This result was comparable with previous studies conduct in Uganda [17] and Venezuela [18], but higher than previous studies conducted in Ethiopia [19–21], and Kenya [22]. This might be due to difference in the socio-demographic factors and lack of awareness on prevention of parasitic infection.

The current high prevalence of *Ascaris lumbricoides* (32.7%) is comparable with the previous study conducted in southern Ethiopia [20], Venezuela [18], and Kenya [22], but higher than studies done in western Ethiopia [21]. This difference may be due to the difference in altitude and awareness in the prevention of parasitic disease.

14.2% of pregnant women were infected with hookworm. This result was higher than studies conducted in southern Ethiopia [23], and Niger Delta regions of Nigeria [24], but lower than previously reported data in western Ethiopia [21].

The prevalence of schistosoma among pregnant women was 17.4%. This result was higher than studies conducted in Southeast Ethiopia [19, 23].

This study identified that intestinal helminth is underestimated public health problem among pregnant women and that socioeconomic factors play an important role in the establishment and spread of the infections in the communities.

Intestinal parasitic infection among pregnant women was determined by age, walking with bare foot, utilization of latrine, hand washing habit, and habit of eating raw vegetables.

Illiteracy increases the odds of intestinal parasitic infection in pregnant women by 2.32 folds higher. This result was in accordance with the previous study conducted in Kenya [25]. This might be due to the health-seeking behavior of literate pregnant women. The odds of intestinal parasitic infection were 2 folds higher in pregnant women living in the rural areas. This finding agrees with finding from south Ethiopia [23]. This is due to the reason that rural pregnant women have less access to the primary healthcare interventions. The odds of intestinal parasitic infections were 3.33 folds higher among pregnant women that didn’t have regular hand washing habit. This finding was in line with previous study conducted in Nigeria [26]. This is due to the reason that proper hand washing practices breaks the chain of transmission for intestinal parasites. Not wearing shoe increases the odds of intestinal parasitic infection by 6.87 times higher. This finding was similar to the previous study conducted in Ethiopia [21]. This is due to the reason that soil-transmitted helminth like hookworm infection will be prevented from entering the susceptible host. Ingestion of raw vegetables increases the odds of intestinal parasitic infection by 2.32 folds higher. A similar previous finding was recorded in Southeast Ethiopia [19, 27]. This is due to the reason that raw vegetables acts as vehicle for transporting intestinal parasites [28–30].

## Conclusion

A high prevalence of intestinal parasites was observed in pregnant women. Walking with bare foot, living in the rural area, illiteracy, age less than 21 years, the absence of proper utilization of latrine, poor hand washing practice and eating raw vegetables were associated factors with a high prevalence of intestinal parasitic infection during pregnancy. Therefore, health educations on hand wash and shoe wearing practice and improvements in sanitary infrastructure could achieve long-term and sustainable reductions in helminth prevalence among pregnant women.

## Abbreviation

*A.lumbricoides*: Ascaris *lumbricoides*
AOR: Adjusted odds ratio
CI: Confidence interval
COR: Crude odds ratio
ML: Milliliters
*P*-VALUE: Probability value
RPM: Revolution per minute
SAF: Sodium acetate- acetic acid-formalin solution
SD: Standard deviation
